# A Nomogram Model of Radiomics and Satellite Sign Number as Imaging Predictor for Intracranial Hematoma Expansion

**DOI:** 10.3389/fnins.2020.00491

**Published:** 2020-06-04

**Authors:** Wen Xu, Zhongxiang Ding, Yanna Shan, Wenhui Chen, Zhan Feng, Peipei Pang, Qijun Shen

**Affiliations:** ^1^Department of Radiology, Affiliated Hangzhou First People’s Hospital, Zhejiang University School of Medicine, Hangzhou, China; ^2^Department of Radiology, The First Hospital of Zhejiang Province, Zhejiang University School of Medicine, Hangzhou, China; ^3^Department of Pharmaceuticals Diagnosis, GE Healthcare, Hangzhou, China

**Keywords:** cerebral hemorrhage/diagnostic imaging, disease progression, computed tomography, stroke, algorithms

## Abstract

**Background:**

We aimed to construct and validate a nomogram model based on the combination of radiomic features and satellite sign number for predicting intracerebral hematoma expansion.

**Methods:**

A total of 129 patients from two institutions were enrolled in this study. The preprocessed initial CT images were used for radiomic feature extraction. The ANOVA-Kruskal–Wallis test and least absolute shrinkage and selection operator regression were applied to identify candidate radiomic features and construct the Radscore. A nomogram model was developed by integrating the Radscore with a satellite sign number. The discrimination performance of the proposed model was evaluated by receiver operating characteristic (ROC) analysis, and the predictive accuracy was assessed *via* a calibration curve. Decision curve analysis (DCA) and Kaplan–Meier (KM) survival analysis were performed to evaluate the clinical value of the model.

**Results:**

Four optimal features were ultimately selected and contributed to the Radscore construction. A positive correlation was observed between the satellite sign number and Radscore (Pearson’s *r*: 0.451). The nomogram model showed the best performance with high area under the curves in both training cohort (0.881, sensitivity: 0.973; specificity: 0.787) and external validation cohort (0.857, sensitivity: 0.950; specificity: 0.766). The calibration curve, DCA, and KM analysis indicated the high accuracy and clinical usefulness of the nomogram model for hematoma expansion prediction.

**Conclusion:**

A nomogram model of integrated radiomic signature and satellite sign number based on noncontrast CT images could serve as a reliable and convenient measurement of hematoma expansion prediction.

## Introduction

Intracerebral hemorrhage (ICH) confers a worse prognosis than ischemic stroke, with an overall fatality rate approaching 40% and neurological disability among the survivors (van Asch et al., 2010; Heit et al., 2017). Based on previous findings, the baseline volume and the location of ICH, intraventricular hemorrhage (IVH), Glasgow coma scale score, and age are strongly associated with the outcomes as clinical predictors (Hemphill et al., 2001). Early hematoma expansion is a greater risk factor for increased mortality and poor functional outcomes, which is independent of other defined clinical correlation factors (Delcourt et al., 2012). A remarkable hemorrhage enlargement by more than 33% volume increase within 24 h after the onset of symptoms occurred in 38% of patients with ICH, as has been reported prospectively (Brott et al., 1997). Therefore, as the only modifiable risk factor, early identification of patients with a potential risk of hematoma growth is crucial for targeted therapeutic strategies.

Recently, different imaging characteristics have been successively reported and paved the way for available prediction of hematoma expansion in clinical routine. The computed tomography angiography (CTA) spot sign, as an independent predictor, has been well established and prospectively validated, which turned out to be of limited sensitivity (Demchuk et al., 2012; Dowlatshahi et al., 2016). Besides that, CTA has not yet been a routine measurement for emergency radiology in some institutions, following nephrotoxicity and allergy problems (Caplan, 2016). Rather, several novel markers based on noncontrast CT (NCCT), including blend sign, black hole sign, swirl sign, island sign, and satellite sign, recently gained attention, mainly focused on the heterogeneity and the irregularity of hematoma (Boulouis et al., 2017; Shimoda et al., 2017; Morotti et al., 2019). The satellite sign, which was easy to recognize and clearly defined, was especially proven to be an independent imaging marker for hematoma expansion prediction (Shimoda et al., 2017; Yu et al., 2017). However, these imaging predictors make qualitative or semi-quantitative analysis studies only and caused inevitable deviation due to subjectivity. More objective quantitative indicators should be defined to approach more veracious results. As a promising quantitative method for heterogeneous studies, radiomic analysis has become a new “’hot spot” in cancer researches (Davnall et al., 2012). Radiomic analysis links quantitative imaging features to clinical findings by using machine learning and statistics analysis methods. Machine learning methods, such as logistic regression, support vector machines, random forest, and Bayesian algorithm, have come into a wider use in the field of radiomics (Lambin et al., 2012; Bi et al., 2018; Shen et al., 2018; Khalaf et al., 2019; Zavecz et al., 2020). In oncology, features have already been carried out to assess intratumor heterogeneity in various tumor types through the analysis of pixel or voxel gray level distribution and degree of coarseness for early diagnosis, preoperative grading, and monitoring responses to therapies for prognosis prediction (Lubner et al., 2017b; Bi et al., 2019). However, limited numbers of studies were found focusing on the nononcologic applications of radiomics (Kotze et al., 2014; Ginsburg et al., 2016; Lubner et al., 2017a).

In this study, we hypothesized that radiomic analysis and quantitative satellite sign can identify the associations between the quantitative imaging features and the hematoma pathophysiology and thus effectively and precisely predict intracerebral hematoma expansion in NCCT images. The aim of this study was to establish a quantitative imaging model to predict hematoma expansion and improve the functional outcomes for patients with ICH. We investigated a nomogram model combined with radiomics and quantitative satellite sign to improve the diagnostic performance in early hematoma expansion prediction.

## Materials and Methods

### Patients

This retrospective study was approved by the Medical Ethics Committee of institution I and II and conducted in accordance with relevant guidelines. Informed consent was waived.

Patients with spontaneous ICH within 6 h since symptom onset and CT recheck within 24 h in between January 2017 and December 2018 were included. The exclusion criteria were the following conditions: (1) patients with ICH secondary to arteriovenous malformation, trauma, aneurysm, tumor, and venous sinus embolism, (2) patients who were receiving anticoagulation treatment, (3) surgery or interventional therapy before the repeat CT scan, (4) image contained severe artifacts, and (5) IVH or subarachnoid hemorrhage is involved. Clinical data were provided by a neurologist, including age, gender, systolic blood pressure, international normalized ratio, time to initial CT scan, activated partial thromboplastin time, and baseline Glasgow Coma Scale score.

### CT Examination, ROI Segmentation, and Imaging Evaluation

The CT scans in the two institutions were carried out on different CT scanners, including a GE LightSpeed VCT 64-slice and a GE Optima 540 16-slice. The same CT scanning parameters were performed with a tube voltage of 120 kV, a tube current of 150–300 mA, field of view of 25 cm, and 512 × 512 acquired matrix. The scan ranged from the skull base to the cranium, with a thickness of 5 mm per layer.

**FIGURE 1:**
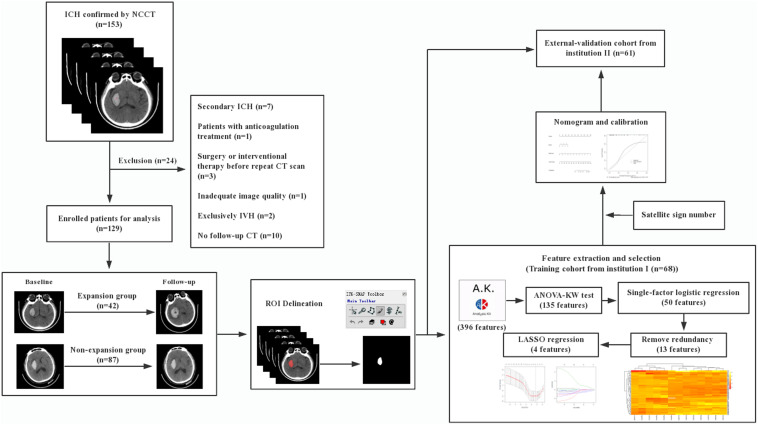
Workflow.

The radiomic workflow is summarized in Figure 1. Patients with a volume increase of more than 33% in the follow-up image within 24 h compared to the initial one were automatically defined as hemorrhage expansion (Connor et al., 2015; Hemphill et al., 2015). ITK-SNAP (Version 3.6.0, UPenn) was performed to segment regions of interest (ROI) on CT images. ROI was delineated manually within the confine of each main hematoma by two neuroradiologists with 10 and 12 years of experience, respectively, and who were blind to the data. Before delineation, intensity normalization by histogram matching was applied to eliminate any difference in technologies using ITK software. All pixel gray levels inside the whole ROI objects were extracted for radiomic analysis. No multiple simultaneous spontaneous ICHs were included in the study (Chen et al., 2016). The two neuroradiologists recorded the location of hematoma, the satellite sign number, and the presence or the absence of swirl sign, blend sign, and black hole sign independently during delineation. The definitions of satellite sign, blend sign, black hole sign, and swirl sign were determined according to Al-Nakshabandi (2001), Li et al. (2015, 2016), and Shimoda et al. (2017).

### Feature Extraction and Selection

All the radiomic features from ROIs were extracted from preprocessed images using the Artificial Intelligence Kit Version 3.0.1.A (Life Sciences, GE Healthcare, United States), with window width 110 and window level 45. Six main categories were involved, including histogram, morphology, gray level co-occurrence matrix, run length matrix, and gray level zone size matrix. Analysis of variance, Kruskal–Wallis test, and single-factor logistic regression analysis were successively carried out for selecting significant features that were highly correlated. By removing the redundancy with a correlation coefficient of more than 0.90, the radiomic features were further optimally elected. In the final step, least absolute shrinkage and selection operator (LASSO) regression was applied to identify the most nonredundant and robust features among the 396 radiomic features from the training cohort in order to improve the class separability and optimize the representation of lesion heterogeneity. With an increase of the value of *λ*, relevant features with non-zero coefficients were selected and these contributed to the final LASSO regression. Meanwhile, the best value of *λ* found by 10-fold cross-validation with a maximum area under the curve (AUC) was used for constructing the regression model. Radscore, which is defined by corresponding non-zero coefficients of the features selected by LASSO, was created by a linear combination of selected features weighted by their coefficients. Respective Radscore was calculated for each patient. Pearson correlation analysis was performed to identify the correlation between satellite sign number and Radscore. The pairwise Pearson correlation coefficients were calculated.

### Radiomics Nomogram Building, Calibration, and External Validation

Both Radscore and the satellite sign number were integrated by a multivariate logistic regression-based radiomic model in the training cohort. Furthermore, a nomogram model was constructed based on a multivariate logistic regression analysis to visually demonstrate the probability of a hematoma enlargement. In addition, predictive models based on Radscore or the satellite sign number alone were also developed. The receiver operating characteristic (ROC) analysis and the AUC were applied to evaluate the discrimination performance on the three models. Along with the Hosmer–Lemeshow test measuring for goodness of fit of the nomogram model, predictive accuracy was assessed *via* a calibration curve in terms of the agreement between the predicted probability of hematoma expansion and the actual one. Then, the constructed model from the training cohort was applied to the external validation cohort. Respective Radscore was also calculated for each patient and further combined with the satellite sign number to validate the nomogram model based on the training cohort. Ultimately, the same process of predictive capability assessment with the ROC analysis and the calibration curve was also carried out in the validation cohort.

Decision curve analysis (DCA) was carried out to evaluate the clinical value of the three models independently on the basis of calculating the net benefit for patients at each threshold probability. By comparing to all strategies or none at all, the best model was elected according to the higher calculated net benefit.

The Kaplan–Meier method was carried out to calculate the survival probabilities. The survival rates were estimated in 30 days. The patients from the two institutions were divided into the expander and the non-expander groups according to the predictive results using the threshold calculated from the training dataset through the Youden Index. Survival was defined as the period from diagnosis to the date of death or the time at which information was last obtained.

### Statistical Analysis

Version 3.3.2 of R software and version 13.0 of SPSS software were used in the statistical analysis. Quantitative variables are shown as mean ± SD. Statistical group comparisons of clinical data were performed by independent-samples *t*-test or *χ*^2^ test where appropriate. Intraclass correlation coefficient (ICC) was analyzed for estimating the reliability of inter-observer agreements, which was defined as good consistency if between 0.75 and 1, fair consistency if between 0.4 and 0.75, and poor consistency if under 0.4. The pairwise comparison of ROC curves was performed using z statistic in MedCalc for Windows, version 19.0.7 (MedCalc Software, Ostend, Belgium). Log-rank test was used to compare survival curves, and the results were considered as significant when *p* < 0.05. The test power (1-β error probability) was calculated by version 3.1.9.7 of G^∗^Power software. The level of statistical significance was set at a two-sided *p-*value < 0.05 for all analyses.

## Results

### Patients Characteristics

As demonstrated in the workflow (Figure 1), between January 2017 and December 2018, a final cohort of 129 patients were selected, and among them, 68 patients from institution I were taken as the training set for the initial prediction model and the other 61 patients from institution II were taken as a prospective independent set for validation.

Tables 1, 2 show the demographic, clinical, and imaging characteristics. No significant difference (*p >* 0.05) was found in all baseline clinical features and in most of the imaging characteristics between expanders and non-expanders in both the training and the validation cohorts. Moreover, only observer one found a statistical significance in the presence of a swirl sign in the training cohort. No other statistically significant difference of the presence of swirl sign blend sign or black hole sign was observed between groups in both cohorts. However, the patients with and without hematoma expansion had an uneven distribution in the satellite sign number with statistical significance (*p* < 0.001).

**TABLE 1:** Baseline demographic information.

Variable	Training set (*n* = 68)			Validation set (*n* = 61)		
	Expander (*n* = 21)	Non-expander (*n* = 47)	*p*-value	Expander (*n* = 19)	Non-expander (*n* = 42)	*p*-value
Age (years)	64.2 ± 14.7	61.2 ± 14.2	0.42	56.2 ± 10.6	57.1 ± 13.5	0.86
Male (%)*	13 (19.1)	31 (45.6)	0.75	6 (20.7)	12 (41.4)	0.98
Admission SBP (mmHg)	168.5 ± 8.8	165.0 ± 11.2	0.21	162.3 ± 9.1	166.1 ± 8.2	0.28
Admission INR	1.5 ± 0.2	1.5 ± 0.3	0.25	1.4 ± 0.3	1.5 ± 0.3	0.51
Time to initial CT scan (h)	3.1 ± 0.9	3.5 ± 1.1	0.21	3.9 ± 1.6	3.9 ± 1.8	0.96
APTT (s)	33.3 ± 4.9	32.0 ± 3.9	0.25	28.3 ± 6.5	29.5 ± 5.0	0.62
Baseline GCS score	12.2 ± 3.8	12.0 ± 3.4	0.84	13.7 ± 3.7	12.3 ± 3.8	0.37

*SBP, systolic blood pressure; INR, international normalized ratio; APTT, activated partial thromboplastin time; GCS, Glasgow Coma Scale. Data are means ± standard deviations. ^∗^Data are the number of patients, with percentages in parentheses.*

**TABLE 2:** Radiological characteristics.

Variable	Training cohort (*n* = 68)			Validation cohort (*n* = 61)		
	Expander (*n* = 21)	Non-expander (*n* = 47)	*p*-value	Expander (*n* = 19)	Non-expander (*n* = 42)	*p*-value
**Observer 1**						
Location*						
Basal ganglia	16 (76.2)	35 (74.5)	0.88	11 (57.9)	31 (73.8)	0.21
Lobar	4 (19.1)	9 (19.2)	0.99	5 (26.3)	7 (16.7)	0.38
Thalamus or brainstem	1 (4.8)	3 (6.4)	0.79	3 (15.8)	4 (9.5)	0.67
Satellite sign number	2.4 ± 1.6	1.0 ± 1.5	<0.001	2.4 ± 1.7	0.7 ± 1.0	0.001
Black hole sign*	8 (38.1)	9 (19.1)	0.10	7 (36.8)	7 (16.7)	0.08
Swirl sign*	7 (33.3)	6 (12.8)	0.04	8 (42.1)	9 (21.4)	0.09
Blend sign*	9 (42.9)	10 (21.3)	0.07	7 (36.8)	9 (21.4)	0.21
**Observer 2**						
Location*						
Basal ganglia	16 (76.2)	36 (76.6)	0.97	11 (57.9)	31 (73.8)	0.21
Lobar	4 (19.1)	8 (17.0)	0.84	5 (26.3)	6 (14.3)	0.23
Thalamus or brainstem	1 (4.8)	3 (6.4)	0.79	3 (15.8)	5 (11.9)	0.69
Satellite sign number	2.5 ± 1.7	1.1 ± 1.4	0.001	2.2 ± 1.1	0.7 ± 0.6	0.003
Black hole sign*	8 (38.1)	8 (17.0)	0.06	9 (47.4)	10 (23.8)	0.07
Swirl sign*	7 (33.3)	7 (14.9)	0.08	8 (42.1)	10 (23.8)	0.15
Blend sign*	7 (33.3)	8 (17.0)	0.13	7 (36.8)	8 (19.0)	0.14

*Data are means ± standard deviations. ^∗^Data are the number of patients, with percentages in parentheses.*

### Reproducibility Analysis

Based on the result of the reproducibility analysis by the two radiologists, 349 out of 396 (88.2%) radiomic features had good consistency (ICC ≥ 0.75) on contour-focused segmentation. The number of features with fair consistency (0.75 > ICC ≥ 0.4) and with poor consistency (ICC < 0.4) was 28 (7.1%) and 19 (4.7%), respectively. Supplementary Table S1 demonstrates in detail the consistency of the four selected radiomic features. For identification of the satellite sign, swirl sign, and blend sign, intraobserver reproducibility analysis was also conducted. The ICC for the satellite sign number was 0.910 (95% CI: 0.855 to 0.945), indicating satisfactory consistency. By contrast, the interrater agreement between the two neuroradiologists for swirl sign, blend sign, and black hole sign was 0.738 (95% CI: 0.575 to 0.838), 0.735 (95% CI: 0.570 to 0.836), and 0.791 (95% CI: 0.700 to 0.854), respectively. Since there is excellent consistency between the two segmentation data as well as the satellite sign number evaluation, data from the neuroradiologist with 12 years of experience were finally submitted for further analysis.

### Radscore and Nomogram Building

**FIGURE 2:**
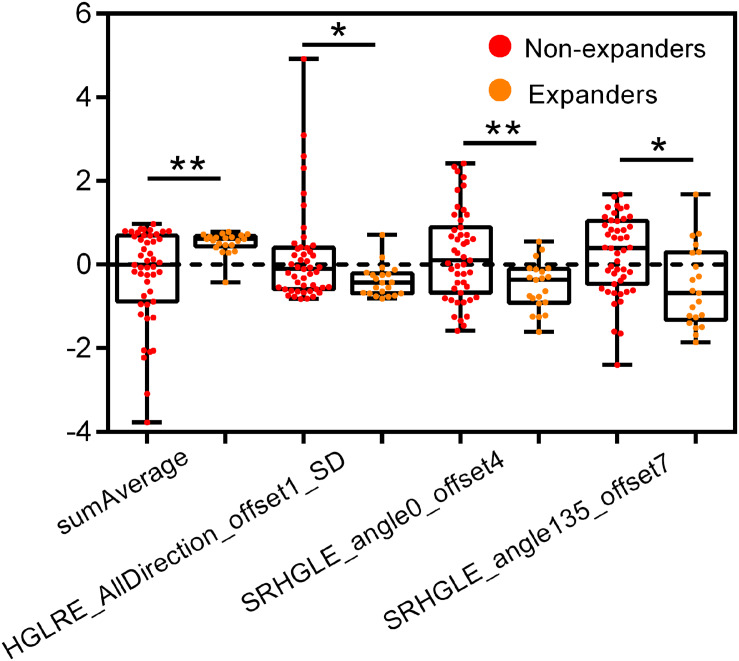
Univariate analysis of four candidate features for hematoma expansion prediction in the training cohort. HGLRE, high gray level run emphasis, SRHGLE, short run high gray level emphasis. *p < 0.01, **p < 0.001.

Supplementary Figure S1A shows the heat map based on feature distribution after redundancy removal. The transparent clustering characteristics between rows implied a high differential capacity of distinguishing between hematoma expanders and non-expanders. Indicated between columns is the clustering identification of the former four features and the latter eight features, respectively. Four features were finally selected by 10-fold cross-validation for ensuring robustness and preventing overfitting (Supplementary Figures S1B,C). The differences of the four candidate features between expanders and non-expanders were all remarkably statistically significant (Figure 2). These features were then constructed by a fitting calculation formula for Radscore.

Radscore=-1.41+(1.29×SumAverage)+(-0.82×Highgray⁢level⁢run⁢emphasis-all⁢direction-offset

1-SD)+(-0.08×Shortrunhighgraylevel

emphasis-angle 0-offset 4)+(-0.61×Short

runhighgraylevelemphasis-angle 135-offset 7)

**FIGURE 3:**
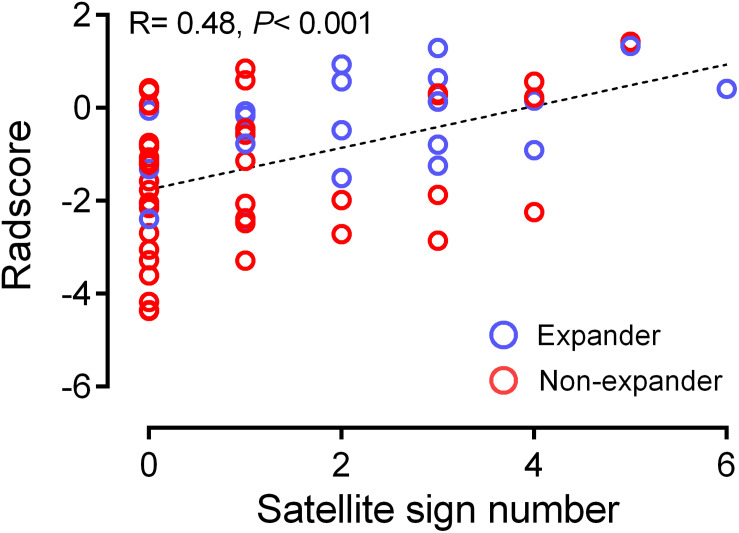
Correlation between satellite sign number and Radscore based on Pearson correlation analysis. The mean absolute correlation was 0.482 (*p* < 0.001, 95% CI 0.272 to 0.649).

**FIGURE 4:**
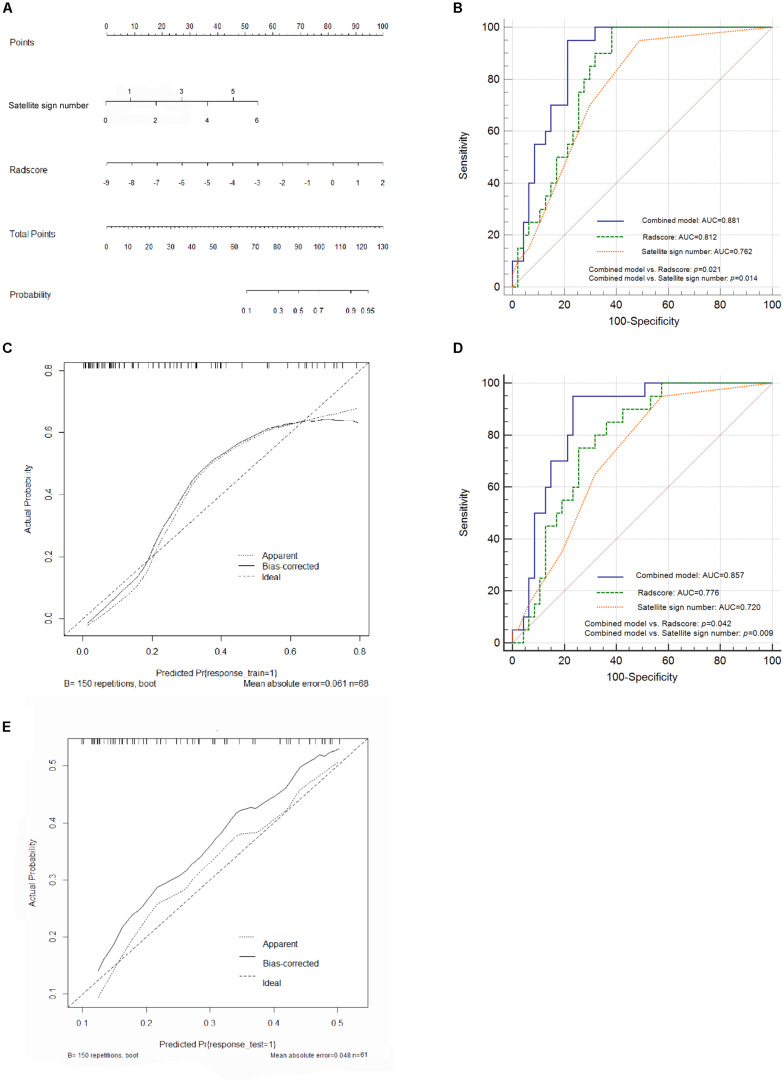
Nomogram construction and performance of the combined model in both cohorts. **(A)** Combined nomogram based on the training cohort. **(B)** Pairwise comparison of receiver operating characteristic (ROC) curves for Radscore, satellite sign number and the nomogram model in the training cohort. **(C)** Corresponding calibration curve in the training cohort. **(D)** Pairwise comparison of ROC curves in the external validation cohort. **(E)** Calibration curve of the nomogram model in the validation cohort.

From the pairwise Pearson correlative analysis, the satellite sign number was observed to be positively correlated to the corresponding Radscore with a correlation coefficient of 0.482 (*p* < 0.001, 95% CI: 0.272 to 0.649) (Figure 3). The multivariable logistic regression analysis was generated on the basis of the Radscore and the satellite sign number. The nomogram model was conducted to visualize the results of the multivariable logistic regression analysis (Figure 4A).

Nomogram=-0.75+(0.82×Radscore)+(0.41×Satellite⁢sign⁢number)

A further validation was carried out through ROC analysis. Compared to the Radscore (0.812, 95% CI: 0.698 to 0.897, sensitivity: 0.992, specificity: 0.617) and the satellite sign number (0.762, 95% CI: 0.643 to 0.858, sensitivity: 0.950, specificity: 0.511) alone, the combination of the two yielded an even better performance in the prediction of hematoma expansion as well as an increased AUC of 0.881 (95% CI: 0.779–0.947, sensitivity: 0.973, specificity: 0.787) in the training cohort (Figure 4B). The nomogram model showed a statistically significant improvement in the pairwise comparison of ROC curves; however, the difference between the Radscore and the satellite sign number has no statistical significance (Figure 4B). Figure 4C illustrates the corresponding calibration curve and the Hosmer–Lemeshow test of the nomogram model in the training cohort (*p* > 0.05). The nomogram model obviously showed a good agreement between the predicted risk and the observed one, indicating a high accuracy of the model in hematoma expansion prediction. The test power (1-β) was 0.99, which verified the reliability and the accuracy of the results (Supplementary Figure S2).

### Performance on the External Validation Cohort

According to the ROC analysis, the nomogram model yielded a higher AUC value (0.857, 95% CI: 0.750–0.931, sensitivity: 0.950, specificity: 0.766) than the Radscore-based model (0.776, 95% CI: 0.657 to 0.868, sensitivity: 0.750, specificity: 0.745) and the satellite sign number (0.720, 95% CI: 0.597 to 0.823, sensitivity: 0.950, specificity: 0.426) in the external validation cohort. Consistent results were shown in the pairwise comparison of ROC curves (Figure 4D). Figure 4E illustrates the calibration curve of the proposed nomogram model based on the validation cohort, which suggested a favorable predictive performance satisfactorily consistent with the ideal curve.

**FIGURE 5:**
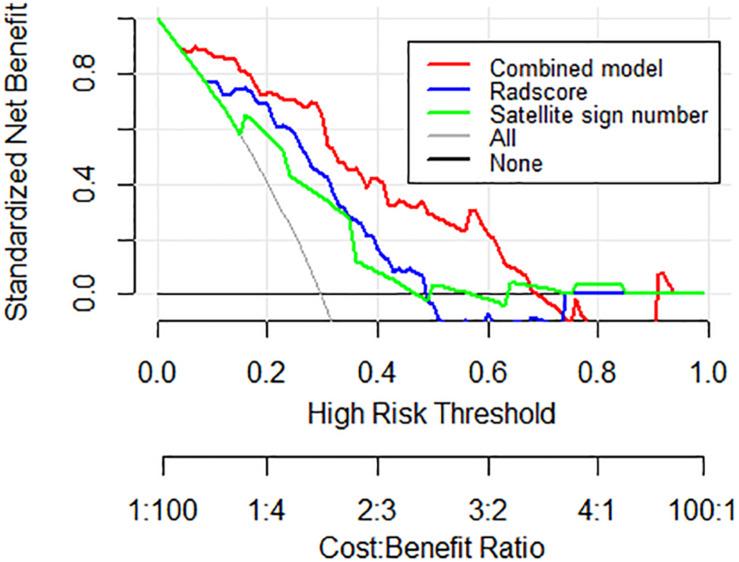
Decision curve analysis for the nomogram model in the external validation cohort. The gray line stands for the assumption that all patients developed hematoma expansion, and the black line represents the assumption that no patient had hematoma expansion. Compared to other models, the highest curve of the nomogram model with more area is the optimal decision making for maximal net benefit in hematoma expansion prediction.

DCA was conducted to assess the clinical utility of the nomogram model (Figure 5). According to the decision curve, the nomogram model (red) demonstrated improved hematoma expansion prediction with more areas shown in the validation cohort compared to that derived from the Radscore or the satellite sign number alone (blue and green).

**FIGURE 6:**
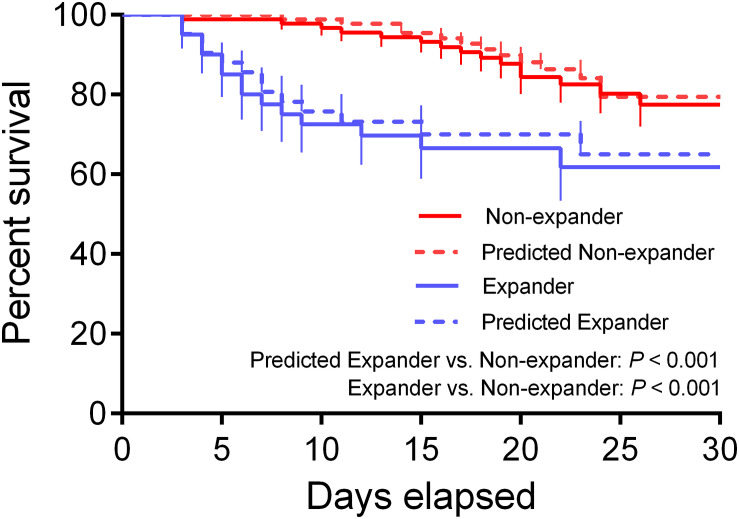
Kaplan–Meier (KM) survival curve for actual and predicted expander and non-expander groups. The KM analysis shows a significant difference between both actual and predicted groups (*p* < 0.001).

The Kaplan–Meier survival analysis showed approximate survival rates between actual subjects and predicted ones. Furthermore, a significant difference was found not only between the actual expander and non-expander groups but also between predicted groups, which suggested the prognostic value of the combined nomogram model (Figure 6, *p* < 0.001).

## Discussion

In this study, we established and validated a nomogram model for early ICH expansion prediction, incorporating four robust radiomic features which were extracted from NCCT and proven to be effective for the classification of expanders and non-expanders and the satellite sign number which was found to be a statistically significant imaging marker for the identification of expanders. The nomogram model achieved a significantly better performance in both training cohort and external validation cohort with a larger AUC value than the model of radiomic signature alone, suggesting the reproducibility and the reliability of the improved model in hematoma expansion prediction.

In the recent years, several imaging markers for assessing the greater risk of ICH expansion in NCCT images have been springing up (Al-Nakshabandi, 2001; Li et al., 2015, 2016; Boulouis et al., 2017; Shimoda et al., 2017). NCCT turned out to be an optimized alternative for patients with ICH. Including blend sign, black hole sign, swirl sign, island sign, and satallite sign, different imaging characteristics based on NCCT have been proposed one after another (Al-Nakshabandi, 2001; Li et al., 2015, 2016; Boulouis et al., 2016b). The imaging biomarkers based on the heterogeneity of hematoma were prone to be impacted by photon noise and to be strongly subjective due to the unclear description and definition, which were dominant factors in reducing interobserver agreement during visual assessment (Park et al., 2019). As a strong evidence for that, the presence of qualitative signs was observed with relatively inferior consistency between two observers, along with indistinctive results between expanders and non-expanders, in our study. Besides that, the evaluation of inter-group statistical significance for swirl sign in the training cohort appeared to be inconsistent between observers. In the study of Xie et al. (2020), no statistical significance was found in the difference of identification capability between the nomogram model and the Radscore-based model for predicting ICH growth, indicating no statistical contribution of qualitative NCCT signs to the nomogram model construction. Meanwhile, inconsistent results of association between NCCT imaging markers and functional outcomes have been found in ICH patients (Boulouis et al., 2016a; Morotti et al., 2017). In this context, instead of defining new imaging predictors, more robust and quantitative characteristics derived from NCCT images were urgently needed to combine with existing markers to contribute to the predictive model for hematoma expansion.

The satellite sign and the island sign shared a similar morphology-based definition, which was less influenced by photon noise during observation (Li et al., 2017; Shimoda et al., 2017). However, the island sign contained a subjective morphological assessment of “islands” that were connected with the “mainland”, which could contribute to the discrepancy between observers (Sporns et al., 2018). Besides that, the lower limit of the island number in the definition made it difficult to transform into a quantitative index. The satellite sign instead had a precise size limitation with complete separation in at least one slice for those “connected satellites,” making the assessment more straightforward, which could be confirmed by the excellent intraobserver agreement and the consistent results between observers found in our study, thus indicating a high reproducibility. The satellites were explained as multifocal active bleeding from peripheral arterioles or reperfusion injury resulting from perihematomal edema, which means the greater the number of satellite was, the higher the probability of hematoma enlargement could be (Fisher, 1971; Shimoda et al., 2017). On top of that, we assumed that satellite sign number detection, a quantitative transformation of the satellite sign, could make imperative complementation for isolated small hematoma that could not be simultaneously included in the radiomic analysis and provide improved risk stratification.

The LASSO regression method has already been widely applied in radiomics-based studies (Huang et al., 2016; Allotey et al., 2019; Wei et al., 2019). The main thrust of LASSO regression is to avoid overfitting by regression coefficient restriction, which shows great strengths when multicollinearity exists. It is suitable for dimension reduction and feature selection in high-dimensional data, especially when the number of features is much higher than the sample size, just in line with the characteristics of radiomic data. Our previous work concentrated on the predictive performance of filtered histogram-based parameters for ICH enlargement (Shen et al., 2018). In this work, we specially focused on amelioration by employing a matrix-based texture extracting approach to improve the category and the quantity of radiomic features. Through an optimized selection from 396 features by the LASSO method, four features including sum average, high gray level run emphasis (all directions), short run high gray level emphasis (angel 0), and short run high gray level emphasis (angel 35) outstandingly surpassed themselves, suggesting their vital role in the prediction model. The sum average measures the relationship between the occurrences of pairs with lower intensity values and higher intensity values. The high gray level run emphasis and the short run high gray level emphasis measure the distribution of high gray level values and the joint distribution of short run and high gray level, respectively. These results indicated the diversity between hematoma expanders and non-expanders on the specific spatial heterogeneity of gray levels within the region of hematoma. What we found was consistent with those of previous studies which selected one or more of these textures as optimum feature for radiomic model construction (Chen et al., 2018; Romeo et al., 2018; Rui et al., 2018). As expected, the Radscore-based model yielded gratifying results in stratifying patients into expanders and non-expanders, with an AUC of 0.812 in the training cohort. In order to establish a more robust nomogram for prediction, the satellite sign number, as described above, was introduced as a promising imaging biomarker for complement. As the results showed, the nomogram model proved to be more effective and reliable than the model of radiomic signature alone in both cohorts, with a satisfactory AUC of 0.881 and 0.857, respectively, suggesting a positive effect of the inclusion of satellite sign number on prediction.

Early hematoma expansion is a critical determinant for both mortality and dependency after ICH onset. As the only modifiable factor in the vast majority of patients, it takes the center stage in therapeutic strategies (Ohwaki et al., 2004; Broderick et al., 2007). Through our study, two experienced neuroradiologists turned out to have rather different results in the observation of swirl sign and blend sign; in spite of that, they reached a high agreement for both radiomic features and the satellite sign number detection. From that, we would say that the nomogram model for ICH expansion prediction was a fast, easy-to-use, and reliable tool, which could be highly efficient and convenient for clinical routine. The model could assess ICH dynamic changes effectively at baseline and facilitate personalized treatment decisions. Owing to that, early medication, intervention, or even decompressive surgery, targeting hematoma growth, could be conducted for those highly suspected expanders as early as possible in clinical practice to improve the long-term prognosis.

There were some limitations in the current research that still need to be further investigated. First of all, it was a retrospective study with a relatively small and imbalanced sample size between expanders and non-expanders in both training and external validation cohorts. Further prospective researches are warranted to expand and balance the sample size and to verify the conclusions. On the other hand, in the process of hematoma segmentation, when it comes to hematoma located in cortical or subcortical regions, it was prone to inaccurate delineation due to partial volume effects. Besides that, the feature extraction software made the displacement vectors 1, 4, and 7 describe the relationship between the gray scale of pixels of the texture as default setting. In light of this, different set points could possibly influence the quantity and the category of radiomic feature extraction; thus, a future radiomic analysis based on various displacement vectors is required. Due to the relatively short follow-up time, the median overall survival for ICH was not available. We will continue to follow up with these patients to secure a more complete prognosis status.

## Conclusion

We have identified and validated a nomogram model of integrated radiomic signature with the satellite sign number based on NCCT images to be a reliable and precise evaluation measurement for ICH enlargement prediction at early baseline. The predictive model could serve as an objective and convenient tool to use for patients with ICH in individualized prediction and treatment decision-making, thus suggesting a great potential for clinical application.

## Data Availability Statement

All datasets generated for this study are included in the article/Supplementary Material.

## Ethics Statement

The studies involving human participants were reviewed and approved by Affiliated Hangzhou First People’s Hospital, Zhejiang University School of Medicine. Written informed consent for participation was not required for this study in accordance with the national legislation and the institutional requirements.

## Author Contributions

WX and QS wrote the manuscript and YS and ZD contributed to the writing process. WX, PP, ZF, and QS analyzed and interpreted the data and prepared the tables and figures. WC, YS, and ZD acquired the data. QS additionally contributed to the conception and the design of the study. All the co-authors read and revised the article.

## Conflict of Interest

PP was employed by company GE Healthcare (China). The remaining authors declare that the research was conducted in the absence of any commercial or financial relationships that could be construed as a potential conflict of interest.
