# Supplementary figures and images for: A Nomogram Model of Radiomics and Satellite Sign Number as Imaging Predictor for Intracranial Hematoma Expansion
Source: Front Neurosci. 2020 Jun 4;14:491. doi: 10.3389/fnins.2020.00491 (PMC7287169; doi:10.3389/fnins.2020.00491)

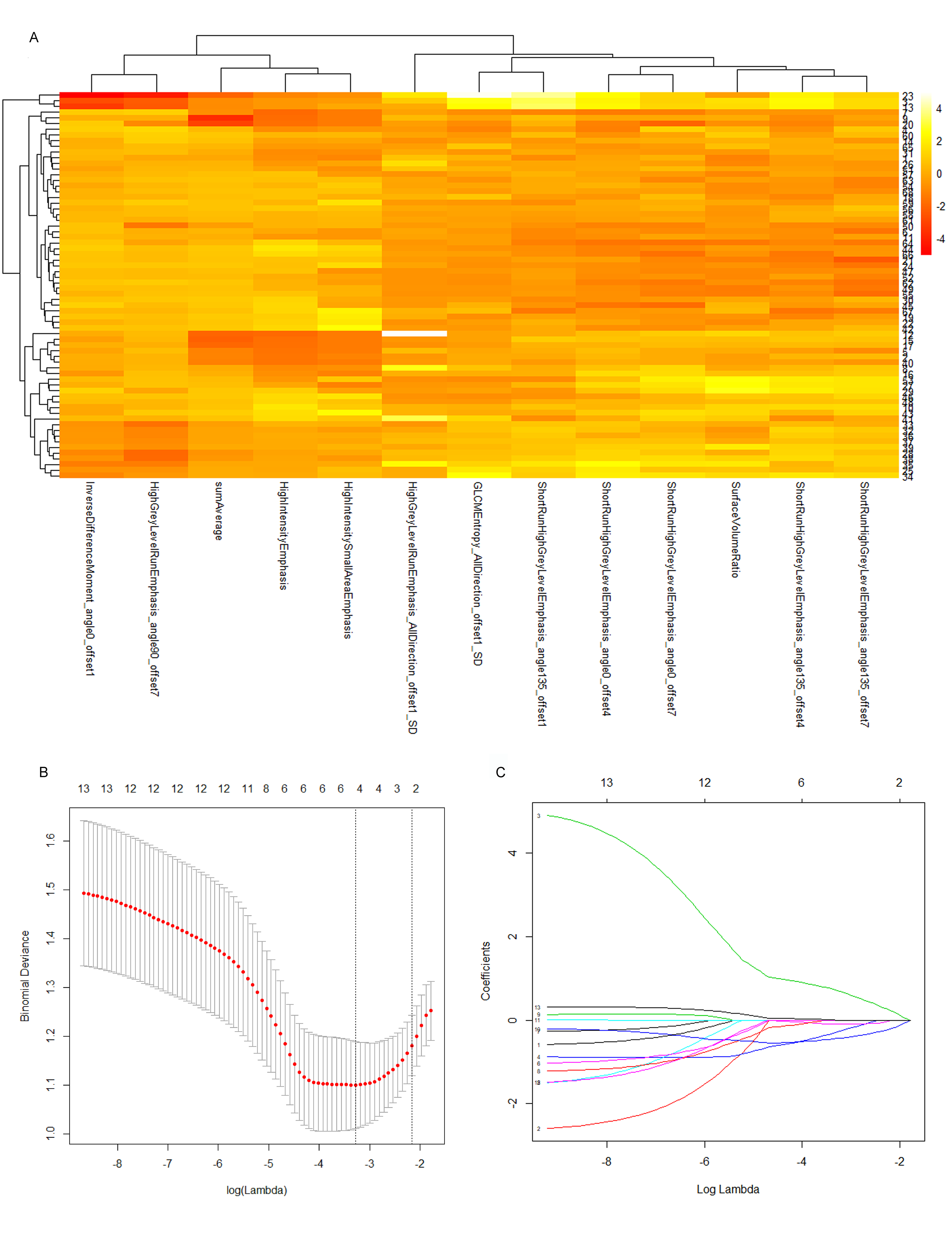

Supplement: Supplementary file 1 [file Image_1.TIF]

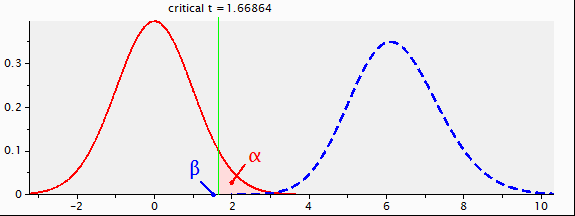

Supplement: Supplementary file 2 [file Image_2.PNG]
