# Supplementary material for: A Nomogram Model of Radiomics and Satellite Sign Number as Imaging Predictor for Intracranial Hematoma Expansion
Source: Front Neurosci. 2020 Jun 4;14:491. doi: 10.3389/fnins.2020.00491 (PMC7287169; doi:10.3389/fnins.2020.00491)
Supplement: Supplementary file 3 [file Data_Sheet_1.doc]

**Supplementary materials**

Table S1: The definition and ICC value of selected texture and imaging features.

| **Name** | **Feature class** | **Descriptions** | **ICC (95%CI)** |
| --- | --- | --- | --- |
| Sum average | GLCM | Sum Average measures overall image brightness | 0.998 (0.995, 0.999) |
| High grey level run emphasis-  all direction-offset 1-SD | GLRLM | Wavelet features derived from High Grey Level Emphasis. High Grey Level Emphasis measures the distribution of high gray level value | 0.934 (0.780, 0.965) |
| Short run high grey level emphasis-  angle 0-offset 4 | GLRLM | Wavelet features derived from Short Run High Grey Level Emphasis. Short Run High Grey Level Emphasis measures the joint distribution of short runs (correlated with coarse textures) and high gray level values | 0.984 (0.960,0.994) |
| Short run high grey level emphasis-  angle 135-offset 7 | GLRLM |  | 0.997 (0.993, 0.999) |
| Satellite sign number |  | The count of small hematoma that separate from the main one on at least one slice with largest transverse diameter < 10 mm and minimal distance between 1 and 20 mm | 0.910 (0.855, 0.945) |
| Presence of Black hole sign |  | Hypoattenuated area encapsulated within the hyperattenuating hematoma with an identifiable border and at least 28 HU difference between the two regions | 0.791 (0.700, 0.854) |
| Presence of Swirl sign |  | Low attenuation area within an extraaxial hyperattenuating fluid collection | 0.738 (0.575, 0.838) |
| Presence of Blend sign |  | Hypoattenuating area adjacent to hyperattenuating region with a clearly defined border | 0.735 (0.570, 0.836) |
| GLCM= grey-level co-occurrence matrix; GLRLM= grey-level run length matrix; HU= Hounsfield unit. | | | |

**Figure Legends**

Figure S1. Feature distribution and selection based on the least absolute shrinkage and selection operator (LASSO) algorithm. (A) The heat map based on feature distribution after redundancy removal. 13 features were respectively elected after removing the redundancy with correlation coefficient more than 0.90. Each row represents every single data from the training cohort with the columns for selected features. (B) Selection of the regulation weight λ by 10-fold cross-validation via minimum criteria to determine the optimal combination of features for Radscore construction. The binominal deviance from 10-fold cross-validation (y-axis) was generated under different log(λ) values (x-axis). The upper x-axis represents numbers of features with non-zero coefficient with a given λ. The left dotted vertical line corresponds to the value of λ with the least binominal deviance. The right dotted vertical line corresponds to the largest value of λ parameter (log(λ min)=-3.27) with the optimal binominal deviance within one standard error of the minimum criteria, at which point the model provided the best fit for data. (C) The LASSO coefficient profile plot was generated against the optimal log (λ) sequence according to (B). Four features with non-zero coefficients were finally selected and contributed to the model construction.

Figure S2. Test power analysis by G*Power software. Power* (1-β err pro) = 0.99.

*The “power” of a statistical test is defined as “1 - β (chance for Type II errors)”, and is the chance of correctly accepting the alternative hypothesis.
